# Distraction arthroplasty compared to other cartilage preservation procedures in patients with post-traumatic arthritis: a systematic review

**DOI:** 10.1007/s11751-018-0305-2

**Published:** 2018-01-23

**Authors:** Jessica C. Rivera, Jason A. Beachler

**Affiliations:** Department of Orthopaedic Surgery, San Antonio Military Medical Center, Joint Base San Antonio, Fort Sam Houston, TX, 78234 USA

**Keywords:** Post-traumatic arthritis, Cartilage, Joint-sparing, Distraction arthroplasty

## Abstract

Post-traumatic arthritis (PTA) is characterized by the deterioration of articular cartilage temporally associated with an articular injury. With a paucity of literature comparing joint preservation techniques, we performed a systematic review of the literature intending to describe and summarize the results of ankle distraction arthroplasty as it compares with studies on tibio-talar microfracture, allograft, and autograft for ankle joint preservation in the post-traumatic population under 50 years of age. Research databases were searched and abstracts screened for relevance on our topic of interest. Abstracts meeting screening criteria with high interobserver reliability underwent full-manuscript review and coding for pertinent citation, study level, treatment, and outcome variables. Outcome variables for patient-reported pain scales, validated outcome measurement tools, radiographic progression, reoperation/re-treatment rates, and complication rates were recorded. Out of 105 unique citations, 10 publications were included. The distraction arthroplasty studies had 36 out of 181 patients requiring reoperation for complications (19.9%), while other joint-preserving procedures studies had 40 out of 177 patients requiring reoperations for complications (22.6%). Clinical outcome scores at mean follow-up time ranging from 2 to 10 years between studies were similar. Reported results for a variety of cartilage preservation procedures, including distraction arthroplasty, are satisfactory and reoperation rates for complication are similar. Limitations in available data and underlying study quality affect synthesis of the results therein. While distraction arthroplasty is an option for cartilage preservation in patients with PTA of the ankle, the technique is highly specialized which may affect the external validity.

*Level of evidence*: III.

## Background

Post-traumatic arthritis (PTA) is characterized by the deterioration of articular cartilage temporally associated with an articular injury [3]. PTA can develop following a high-energy joint injury, such as an intra-articular fracture, or following a lower energy injury such as a sports-related ligament rupture [2, 3]. Because both athletic injuries and traumatic injuries are most common among young demographics, PTA typically affects young individuals. This is in contrast to degenerative arthritis which occurs as a result of normal age-related cartilage loss, manifesting in much older individuals. The ankle is particularly sensitive to articular injury, in time leading to the development of PTA. Approximately 70% of ankle replacement surgeries are performed in patients with PTA rather than degenerative arthritis [13]. Ankle arthritis is also particularly debilitating, resulting in functional deficits comparable to end-stage hip arthritis, congestive heart failure, and end-stage kidney disease [8, 18].

There is currently no consensus on how best to treat PTA of the ankle. However, there are several surgical options aimed at either treating pain associated with the deteriorating joint or attempting to repair or preserve the remaining cartilage. One historically reliable method is the ankle arthrodesis whereby the degenerative tibio-talar joint is fused [19]. While this method does reduce the pain associated with motion at a degenerative joint, the arthrodesis results in loss of range of motion and can negatively affect adjacent joints of the hind- and mid-foot. In an attempt to preserve motion and spare adjacent joint disease, ankle joint arthroplasties are now marketed and often compared to arthrodesis in terms of patient-reported and functional outcomes [19]. However, both of these common procedures are ablative to the native joint, an option which may not be desirable as a long-term solution in a young patient with PTA. Furthermore, both the arthrodesis and total joint arthroplasty are best indicated in advanced stages of arthritis which fails to address the clinical scenario where isolated traumatic injuries do not immediately cause the entire joint to deteriorate. In such cases, especially in a younger patient, a joint preservation procedure may be more desirable.

There are several surgical joint preservation options. Cartilage marrow stimulation procedures (microfracture), autografts, and allografts may be used to attempt repair of isolated cartilage defects of the tibial plafond or the talus, and block allografts may be used to replace a larger portion of talus [5, 23]. A relatively new surgical procedure intended for joint preservation, distraction arthroplasty, does not use any graft material. This procedure employs an external fixator which crosses the ankle joint and applies a distraction force across the tibio-talar articulation [6, 12, 17]. The theory behind distraction arthroplasty is that it allows for the reparative potential of the joint by removing mechanical stress. Among joint preservation options, distraction arthroplasty offers several theoretical advantages and is less invasive than “traditional” cartilage repair procedures using autograft or allograft.

In the current literature on ankle joint preservation procedures, a recent review (2012) on the clinical indications for distraction arthroplasty concluded that the present literature does not support any particular surgical indication for the procedure [20]. For PTA specifically, the authors indicated that due to the poor quality of published reports, isolated to relatively small case series and expert opinion, a recommendation for use of distraction arthroplasty to treat PTA could not be supported. A systematic review (2015) of six cartilage repair procedures likewise concluded there were poor study methods and a lack of sufficient level of evidence to make a recommendation for any of the procedures [15]. Because the available reviews focus on the level of evidence supporting surgical indications for individual procedures rather than patient outcomes, there are no reviews examining the available published outcomes for distraction arthroplasty compared to other preservation procedures.

In spite of the present lack of high-level evidence, a comparison of various cartilage repair procedures and distraction arthroplasty would fill a knowledge gap for the surgeons treating ankle arthritis patients. This is especially true for young PTA patients for whom the cumulative disability-adjusted life years are potentially substantial and procedure selection must be as well informed as possible. The purpose of this systematic review is to describe and summarize the results of intervention studies of distraction arthroplasty used to treat post-traumatic arthritis in patients less than age 50 years of age compared to intervention studies on tibio-talar microfracture, allograft, and autograft for ankle joint preservation. The outcomes to be used for comparison are patient self-reported outcomes including American Orthopaedic Foot and Ankle Society (AOFAS) score, frequency of reoperation, and progression of arthritis as per the Kellgren–Lawrence grade.

## Materials and methods

### Inclusion and exclusion criteria

Eligible studies included all original articles on human patients with ankle arthritis treated with either distraction arthroplasty, tibio-talar microfracture, allograft, or autograft. Basic science, animal, and cadaver studies were excluded. Studies were not restricted on patient demographics except that a portion of the patient population had to be younger than age 50. Studies had to include any portion of patients treated for PTA of the ankle, but were not required to have PTA patients as their only cohort representatives. Articles reporting only on the total ankle arthroplasty or ankle fusion were excluded. Study types included were case reports, case series, case–control studies, cohort studies, and randomized trials. Included articles had to report at least one clinical outcome via either a pain scale, validated patient-reported outcomes, clinician assessment of radiographs, or reoperation rate. There was no limitation on date of publication. Articles not published in English as full text were not excluded if an informative abstract in English was available including pain and outcome results with standard deviations.

### Search strategy

MEDLINE (Ovid) and PubMed (National Library of Medicine) were searched with the assistance of a health sciences librarian experienced in formatting search strategies for systematic reviews. Concepts formulating the search included: ankle arthritis, ankle joint injury, cartilage injury, distraction arthroplasty, cartilage preservation, microfracture, allograft, and autograft. Resultant articles underwent screening of their bibliographies for potentially pertinent publications not identified in the database searches. Citations were stored in RefWorks (Proquest^®^) and screened for duplicates. Search strategies, results, and abstracts chosen for screening were managed using Microsoft^®^ Excel workbook designed for systematic reviews [14].

### Screening procedures

Two authors (**, **), both orthopedic surgeons, blinded to each study’s authorship and journal of publication independently screened all titles and abstracts for exclusion criteria. Screener agreement was excellent (Cohen’s kappa = 0.96). Abstract data were then complied in the Microsoft^®^ Excel workbook, and any disagreement regarding possible study inclusion based on the abstract screening was reconciled, erring on the side of full-text review [22].

### Full-text review procedures

Each full-text article, or included abstract where applicable, was fully reviewed and coded for pertinent citation, study level, treatment, and outcome variables by the primary author. Citation variables included basic study information listed in the Ovid/Medline or PubMed citation, what type of source was used, and if the study was related to another reviewed article. Study-level variables included information on the study design and execution, including eligibility criteria. These included diagnoses represented with an eligibility requirement that at least a subset of subjects will be diagnosed with PTA. Study design, location, years of performance, and sample characteristics such as age, sex, and practice environment were included for demographic and external validity purposes. Study conduct descriptors such as sampling method, procedure selection methods, comparison groups, and basic statistical plan were recorded to address internal validity. Where applicable, funding source(s) were also recorded.

### Coding procedures

Treatment and follow-up variables were recorded to indicate which procedures of interest were studied, indicating if a study contained subjects who underwent multiple procedures. The length of follow-up, length of time distraction arthroplasty subjects were in their external frames, and whether or not differential follow-up occurred were recorded. The “frame time” was considered because this treatment duration overall can skew the total follow-up time. This is because subjects who undergo any of the other procedures (microfracture, autograft, or allograft) essentially begin their follow-up period immediately following surgery. Subjects who undergo distraction arthroplasty, however, undergo a surgery to apply the distraction frame which is worn for a period of time. The frame is then removed at a second procedure, and it is after this frame removal procedure that the follow-up in terms of outcome measures begins. The implication of this is a microfracture patient who is studied at 12 weeks following surgery cannot be compared to a distraction arthroplasty patient who is 12 weeks from study enrollment, but may only be 8 weeks out of his or her distraction frame. Noting “frame time” and differential follow-up was used to correctly interpret the follow-up periods reported per study.

Outcome variables were recorded per procedure for patient-reported pain scales, validated outcome measurement tools including the American Orthopaedic Foot and Ankle Score (AOFAS) and Ankle Osteoarthritis Score (AOS), radiographic progression per surgeon interpretation of the Kellgren–Lawrence (K/L) grade, reoperation/re-treatment rates, and complication rates. For the scoring criteria, the final scores and some indication of change from baseline were recorded as the data allowed. Time points for the measurements were also recorded.

One reviewer (**) worked independently to code all the articles included. A second reviewer (**) independently coded a random sample of articles blinded to the study authorship and author institution(s) to pilot-test the codebook and coding. Both coders are orthopedic surgeons familiar with the described surgical techniques and the measurement of outcomes included. After establishing the reliability of the coding process, the review of all articles again blinded to study authorship and author institution(s) ensued. Results for pre- and post-procedure pain scores, patient-reported outcome scores, and surgeries, and complications were compiled among the studies reporting results for distraction arthroplasty and for the studies reporting results for the other procedures.

## Results

**Fig. 1:**
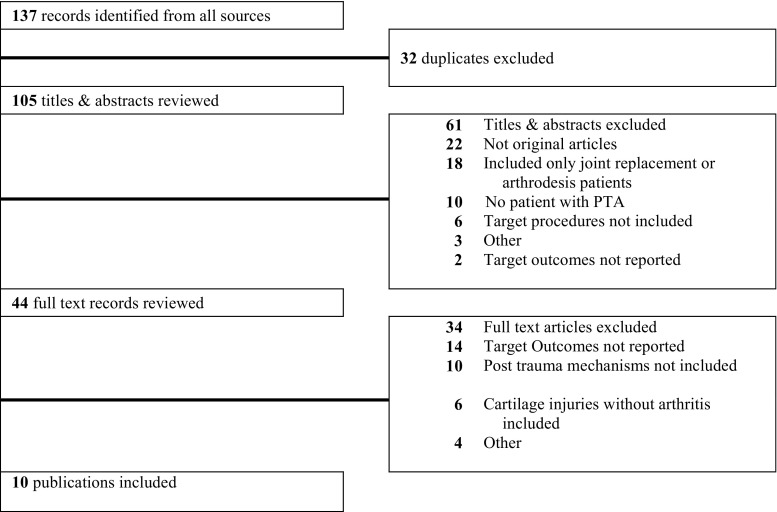
PRISMA chart

The Ovid MEDLINE and NLM PubMed searches yielded 105 unique citations. Based on the exclusion criteria, 61 titles/abstracts were excluded and 44 retained for full-text review. No foreign language abstracts were included in the full review due to lack of usable data included within the English version abstracts. After full-text review, an additional 34 articles are excluded because of lack of reporting targeted outcomes, lack of inclusion of post-traumatic-specific mechanisms leading to arthritis, lack of arthritis in the included patients despite post-traumatic injury to cartilage, and other reasons (Fig. 1).

Included studies represented subjects with mean age between 24 and 43 years of age. No studies included minors in their data. All studies had a male predominance. While not all studies specified what subset of subjects was treated for post-traumatic conditions, five of the 10 studies included only subjects with post-traumatic arthritis. Study designs ranged from prospective randomized trial design to retrospective studies. Of the study with subjects only treated with distraction arthroplasty, the mean follow-up time was inclusive of the frame time for each subject as total frame time was reported on in one study. Mean length of follow-up studies was between 2 and 10 years (Table 1).

**Table 1:** Included study details

References	Sample size	Subject mean age (years)	Subjects with PTA	Study design	Procedures represented	Mean length of follow-up
Becher et al. [1]	45 (25 males)	40 ± 14	31 69%	Prospective observational	Microfracture	5.8 ± 2.0 years
Buda et al. [4]	56 (37 males)	35.6	56 100%	Prospective observational	Autograft	36 months
Giannini et al. [7]	26 (18 males)	34.9 ± 7.7	26 100%	Prospective observational	Bipolar osteochondral allograft	40.9 ± 14.1 months
Gobbi et al. [9]	21 (14 males)	24 27.8	Not specified	Prospective randomized	Microfracture, autograft	53 months
Jeng et al. [10]	29 (15 males)	41	Not specified	Prospective observational	Bipolar osteochondral allograft	2 years
Marijnissen et al. [11]	105 (67 males)	42.7 ± 9.8	Not specified	Prospective randomized/observational	Distraction arthroplasty	2 years
Nguyen et al. [14]	29 (15 males)	41.5 ± 9.1	29 100%	Observational cohort	Distraction arthroplasty	8.3 ± 2.2 years
Ploegmakers et al. [16]	22 (14 males)	37 ± 11	19 86%	Retrospective prospective cohort	Distraction arthroplasty	10 ± 2.5 years
Saltzman et al. [17]	36 (24 males)	42.4 (18–59)	36 100%	Randomized (fixed versus motion)	Distraction arthroplasty	104 weeks
Tellisi et al. [21]	25 (16 males)	43	25 100%	Retrospective	Distraction arthroplasty	30 months Frame time 12 months

All included studies provided outcomes on pain, patient-reported outcome score, or both. Pain and outcomes scores were reported using variable scales. The pain scores reported include the visual analogue scale (VAS) scores as well as pain components of clinical outcome scores. Of the clinical outcomes reported, the HSS, the AOFAS, and the AOS are all used in the literature. HSS (Hannover Scoring System) rates patient’s complaints and functional status using a 20-question report. The AOFAS (American Orthopaedic Foot and Ankle Society) is a 100-point scale integrating pain, function, and alignment. This scoring system requires clinical measurements on both examination and radiographs. The AOS (Ankle Osteoarthritis Scale) is a self-assessment tool that measures disabilities related to ankle pain. All three of these tools have been used in the literature and have been validated. However, they are not interchangeable and cannot necessarily be translated into one another.

The progression of arthritis was reported in four studies using various methods for determining OA grade with no studies reporting K/L grade and two of the four studies reporting on MRI results rather than plain radiographs. Additional treatment was poorly specified; however, all but one study reported on revision surgeries. The revision surgery rate ranged from 8.0 to 48.3%. Combined, the distraction arthroplasty studies had 36 out of 181 patients requiring reoperation for complications (19.9%) and the other procedures studies had 40 out of 177 patients requiring reoperations for complications (22.6%) (Table 2).

**Table 2:** Included study reported outcomes

References	Pain pre/post	Outcome score pre/post	OA progression	Additional treatment	Additional surgery
Becher et al. [1]	VAS score 6.5 ± 2.3 2.4 ± 2.8**	HSS (function) 3.6 ± 2.3/7.2 ± 2.8**	Not given	Not specified	4/45 8.9%
Buda et al. [4]	Not given	AOFAS 52.3 ± 14.2/77.8 ± 18.3	15/56 26.8%	Not specified	16/56 28.6%
Giannini et al. [7]	Not given	AOFAS 26.6 ± 6/77.8 ± 8.7**	26/26 100%	3/26 11.5%	3/26 11.5%
Gobbi et al. [9]	Not given	AOFAS 3.8/83.8** 31.1/85.4**	Not given	1/21 4.8%	3/21 14.3%
Jeng et al. [10]	AOFAS (post) “success” only: 31	AOFAS (post) “success” only 84 (71–96)	6/29 20.7%	Not specified	14/29 48.3%
Marijnissen et al. [11]	AOS 67.1 ± 15.2/38.2 ± 23.8	AOS 68.3 ± 15.1/35.9 ± 23.0	Not given	2/105 1.9%	16/105 15.2%
Nguyen et al. [14]	Not given	AOS 60.7 ± 12.2/34.4 ± 24.0	Not given	Not specified	13/29 44.8%
Ploegmakers et al. [16]	Van Valburg 78 ± 3%/30 ± 5%**	AOS 67 ± 6/25 ± 6*	Not given	1/22 4.5%	5/22 22.7%
Saltzman et al. [17]	Not given	AOS 63.14 ± 11.88 (motion) 62.77 ± 13.23 (fixed)/34.64 ± 19.12 (motion) 54.29 ± 21.74 (fixed)	Not given	Not specified	Not specified
Tellisi et al. [21]	AOFAS 15/31	AOFAS 55 (29–82)/74 (47–96)*	2/25 8.0%	Not specified	2/25 8.0%

*p < 0.05; **p < 0.001

## Discussion

Individual studies each reported improvements in pain, patient-reported outcomes, or both following the studied procedures. Follow-up after distraction arthroplasty results and results from the other procedures indicated similar reoperation rates for complications. These findings suggest that surgeons considering distraction arthroplasty can expect similar pain and patient-reported outcome results and similar reoperation rates compared to other cartilage preservation procedures excluding the mandatory operation to remove the frame itself. Advantages therein are that distraction arthroplasty does not preclude the use of other procedures in the future and may even be used in combination with the other cartilage preservation options discussed (e.g., microfracture plus distraction). Given the natural history of ankle arthritis, it is expected that all patients, regardless of cartilage preservation procedure will deteriorate. Distraction arthroplasty appears to be a reasonable option to provide a good percentage of patients a satisfactory period of time of improvement. However, because distraction arthroplasty is somewhat of a specialty technique, it would be important for future studies to include analysis of surgeon training and experience with this technique as they relate to patient outcomes.

The search strategy was intended to be comprehensive of the peer-reviewed literature over the breadth of date ranges during which distraction arthroplasty specifically has been developed. By focusing on cartilage preservation options, articles focused entirely on joint ablative procedures such as total ankle arthroplasty and tibio-talar fusion were eliminated. This is because clinically joint preservative and joint ablative procedures cannot be compared from the prospective of the longevity of the patient’s native cartilage. Non-English literature with available English abstracts was not excluded from screening, assuring that research performed on distraction arthroplasty specifically was not overlooked if published primarily in Europe where much of the early literature on other Ilizarov techniques originated. Limitations of the search strategy include the non-availability of full-text articles in languages other than English. Additional search of Embase could be conducted to assure coverage of European publications as well as an additional search of non-peer-reviewed publications. Original articles were expected to be of low quality of evidence which then also had implications for the quality of data available for review. However, thoughtful examination of the cumulative published population on distraction arthroplasty where specific outcomes were reported will add to the current understanding of this relatively new procedure. Additionally, many of the articles commented on “revisions,” but there was inconsistency in what that entailed. It would be preferable to know the exact number of patients going on revision to fusion or arthroplasty versus those that were revised with an additional joint-sparing procedure. Unfortunately, the literature was not precise enough to make this analysis part of our data.

Strengths of the employed coding protocol included considerations for the various potential procedures and capturing the outcome variables per procedure. This is necessary if comparisons are to be made between the procedures. The protocol also included consideration of the external and internal validity of the included studies in planning coded variables as prior reviews on distraction arthroplasty emphasize the specialized nature of the procedure and the low level of evidence available. Limitations of the coding protocol included a single coder for all articles and the inherent inability to completely capture the clinical decision-making process. With few randomized trials available, the majority of the literature comprised level 3–5 evidence where individual providers can instill a large amount of selection and performance bias into the their study designs. While the coding protocol did distinguish between study design-level decision making versus surgeon discretion, the coding was unable to fully capture how subjects were selected for the included studies. Finally, the coding protocol did not account for studies which reported MRI outcomes rather than radiographic outcomes. This limited the interpretation for this target variable as plain radiograph and MRI results cannot be meaningfully compared.

There are additional limitations to considering pooled results for these studies. First of all, complications typical of distraction arthroplasty and the other cartilage preservation procedures are expected to be very different. Ilizarov techniques have added potential complications such as pin tract infections and neurologic palsies from traction which could necessitate additional medical therapy and even surgical revision of the distraction frame or frame program [6, 21]. These complications arguably are not comparable to the complications typical for the other included procedures which are not very different than the typical risk of out patient orthopedic surgery. Secondly, distraction arthroplasty and other Ilizarov and circular frame techniques are often performed by specialty-trained surgeons often at centers known for limb reconstruction services. Except perhaps the bipolar ankle autograft, the other cartilage preservation procedures included are not outside the purview of a typical general orthopedic practice. Therefore, the results reported for distraction arthroplasty have poor external validity to general practice and many non-Ilizarov trauma and foot and ankle practices.

## Conclusion

Reported results for a variety of cartilage preservation procedures, including distraction arthroplasty, are overall satisfactory, and reoperation rates for complication following distraction arthroplasty and other “traditional” procedures appear to be similar. Limitations in available data and underlying study quality in terms of levels of evidence may affect synthesis of the results therein. While distraction arthroplasty is an option for cartilage preservation in patients with PTA of the ankle, the technique is highly specialized, which may affect the external validity of case series from specialized Ilizarov centers.
